# P-1519. Susceptibility of Clinical Non-*Morganellaceae* Enterobacterales Isolates from Bloodstream and Respiratory Tract Infections to Imipenem/Relebactam and Comparators: SMART United States 2020-2022

**DOI:** 10.1093/ofid/ofae631.1688

**Published:** 2025-01-29

**Authors:** Mark G Wise, C Andrew DeRyke, John Esterly, Karri A A Bauer, Fakhar Siddiqui, Katherine Young, Mary Motyl, Daniel F Sahm

**Affiliations:** IHMA, Schaumburg, Illinois; IHMA, Schaumburg, Illinois; Merck & Co., Inc., Rahway, New Jersey; Merck & Co, Inc, Kenilworth, New Jersey; Merck & Co., Inc., Rahway, New Jersey; Merck, Rahway, New Jersey; Merck, Rahway, New Jersey; IHMA, Schaumburg, Illinois

## Abstract

**Background:**

Enterobacterales with multidrug-resistance (MDR) or difficult-to-treat resistance (DTR) present clinicians with limited treatment options. Imipenem/relebactam (IMR) is a combination of imipenem with relebactam, a β-lactamase inhibitor of class A and C β-lactamases. We examined the activity of IMR and comparators against isolates of non-*Morganellaceae* Enterobacterales that were the etiological agents of bloodstream and respiratory infections in the United States.

**Figure:**
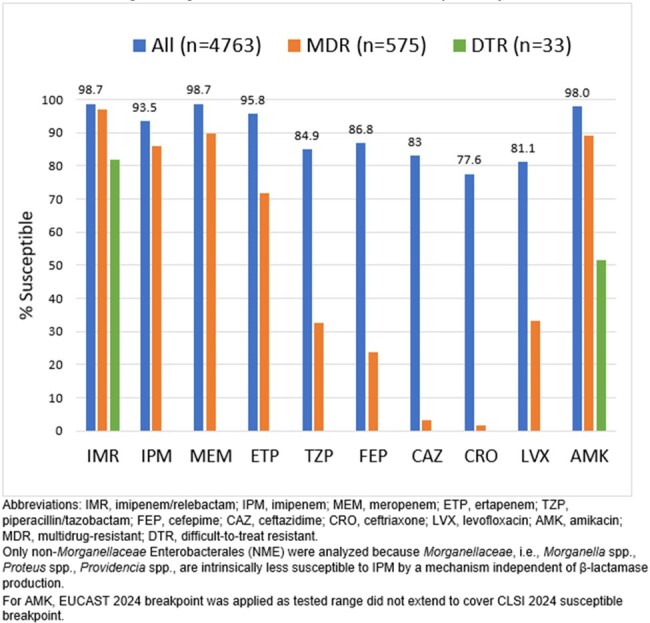

**Methods:**

In 2020-2022, 26 clinical labs in the U.S. participated in the global SMART surveillance program each collecting up to 250 consecutive gram-negative pathogens per year. Only isolates from patients with bloodstream and respiratory tract infections were included in this study. MICs were determined using CLSI broth microdilution and interpreted with 2024 CLSI breakpoints. MDR was defined as resistance to ≥3 sentinel agents (amikacin, aztreonam, cefepime, ceftazidime, colistin, imipenem, levofloxacin, and piperacillin/tazobactam). DTR phenotypes were defined by isolates nonsusceptible (intermediate or resistant) to all β-lactams (including aztreonam, ceftazidime, cefepime, imipenem, meropenem, piperacillin-tazobactam), as well as fluoroquinolones (levofloxacin).

**Results:**

Among 4,763 collected NME isolates, 575 (12.1%) were MDR and 33 (0.7%) were DTR. *Escherichia coli* (n=1695) and *Klebsiella pneumoniae* (n=958) were the most common species collected and their MDR rate was 12.2% and 13.5%, respectively, while their DTR rate was 0.1% and 1.8%, respectively. Against the full collection, IMR and meropenem were the most active agents, each inhibiting 98.7% of the population (Figure). Ertapenem, amikacin and imipenem alone were also active, inhibiting >93%. IMR retained its activity versus the MDR subset, as 97.0 were interpreted as susceptible, the highest percentage among comparator drugs. IMR also showed activity against the challenging DTR subsets with 81.8% susceptible, approximately 30 percentage points higher than amikacin.

**Conclusion:**

Based on these *in vitro* data, IMR appears to be an excellent therapeutic choice for use against NME infections from blood and respiratory tract sources, including those identified as MDR and DTR.

**Disclosures:**

**Daniel F. Sahm, PhD**, Pfizer, Inc.: Advisor/Consultant

